# Automatic Suppression Method for Water Surface Glints Using a Division of Focal Plane Visible Polarimeter

**DOI:** 10.3390/s23177446

**Published:** 2023-08-26

**Authors:** Meishu Wang, Su Qiu, Weiqi Jin, Jie Yang

**Affiliations:** MOE Key Laboratory of Optoelectronic Imaging Technology and System, Beijing Institute of Technology, Beijing 100081, China

**Keywords:** polarization imaging, division of focal plane polarimeter, water surface image processing

## Abstract

To address the problem of water surface detection imaging equipment being susceptible to water surface glints, this study demonstrates a method called De-Glints for suppressing glints and obtaining clear underwater images using a division of focal plane (DoFP) polarimeter. Based on the principle of polarization imaging, the best polarization angle and the image corresponding to the minimal average gray level of each pixel are calculated. To evaluate the improvement in image quality, the index *E* was designed. The results of indoor and outdoor experiments show that the error of the angle calculation of this method is within 10%, and the minimum error is only 3%. The *E* index is positively improved and can be relatively improved by 8.00 under the interference of strong outdoor glints, and the method proposed in this paper shows a good adaptive ability to the dynamic scene.

## 1. Introduction

For human survival, a good water environment is indispensable. Effective management of the water environment requires regular and continuous surveillance of the surface environmental conditions of rivers, lakes, and seas in order to obtain the distribution of floating objects or aquatic organisms on the water surface, underwater plants, and pollutants discharged underwater. Sensors [1,2,3], hydrological remote sensing [4], imaging detection [5,6,7], and other technologies are effective technical means to achieve these ends. Imaging detection techniques provide intuitive water conditions in the form of non-contact telemetry images compared to sensor detection techniques and result in the better identification of large volumes of solid pollutants on the water surface. Compared with hydrological remote sensing, such approaches are more suitable for small-scale clear monitoring near the surface of the water. Different types of cameras have been employed as important monitoring tools. Some cameras are arranged on traditional water surface monitoring boats; meanwhile, along the river, fixed monitoring cameras or unmanned aerial vehicle dynamic cameras are increasingly becoming important means of monitoring the water for rapid and effective surveillance.

The monitoring of water environments is arduous because of the reflection of sunlight. On clear days, the sunlight reflected from the water surface often produces strong glints, which can cause large-area saturation of the camera, the loss of pixel information, the erroneous judgment of underwater target detection, and other strong radiation interference problems. In these cases, errors are often introduced in ocean water color remote sensing, surface target surveillance, and other imaging applications [8]. Therefore, eliminating or minimizing the influence of water surface glints significantly affects image quality [9,10,11].

Glints are produced by the reflection of strong sky or solar radiation, which exhibit remarkable polarization features [12,13], related to the observation and solar conditions [14,15,16,17]. It has long been common knowledge that glints can be reduced by using polarized imaging [18,19]. A polarizer is an essential accessory in photography [20]. Photographers can obtain polarized images by rotating the polarizer attached in front of the camera. Such systems are referred to as division of time (DOT) polarimeters [21]. When using DOT polarimeters, the transmission direction of the polarizer should be perpendicular to that of the glints. In this state, information on intensity can be obtained with minimal glint impact in a single polarization direction [22,23]. In line with DOT polarimeters, orthogonal polarimetric imaging modes have been developed to better suppress glints by capturing images in the best and the worst detection directions. Glint suppression methods based on DOT polarimeters have achieved remarkable results in static scenarios. However, in practical engineering applications, their development has been limited by their complex construction. Accurate and dynamic adjustments of position are challenging because of the constantly changing platform and detection position. This renders the dynamic optimization of the direction of the best detection angle necessary. Moreover, the relative inclination of the water surface within the detection field of view is also inconsistent.

These issues have been well addressed since the introduction of the fully polarized imaging technique. Fully polarized imaging systems can simultaneously obtain polarization images of the scene in three to four directions, which can be used to not only obtain the image of any detection direction of the scene, but also calculate polarization information such as the degree of polarization (DoP), angle of polarization (AoP), and Stokes vector. Typical polarization imaging modes such as the division of amplitude [24], division of aperture [25], and DoFP polarimeters [26,27] have been proposed and developed extensively, among which the DoFP polarimeter is the most compact. It couples an array of 2 × 2 micro polarizers as a polarization unit to the corresponding CMOS detector image pixels. Thus, the intensity of the four detection directions can be obtained dynamically. The DoFP polarimeter is a current research hotspot and has made important advances in applications such as food testing [28] and biomedical applications [29]. It is expected to achieve glint suppression by individually calculating polarization information according to the fluctuation of the water surface in different areas, thus rendering it particularly suitable for environmental detection in scenes containing water surfaces or shallow water areas. Meanwhile, the DoFP polarimeter retains the advantages of the DOT polarimeter in suppressing water surface glints, while featuring real-time performance. However, there is still insufficient research on how to quickly achieve glint suppression based on real-time polarization images.

In this study, the dynamic adaptation capability of the DoFP polarimeter is utilized to study the automatic suppression method of water surface glints in water environmental observation. Section 2 of the paper presents a novel polarization-based suppression method for a DoFP polarimeter based on a Stokes vector polarization imaging model. Furthermore, Section 3 introduces the experiments and Section 4 presents a comparison of the proposed method using DoFP with previous methods using DoT polarimeters, before finally analyzing the results.

## 2. Methods

Based on the Stokes vector polarization imaging model, we analyzed the conventional method by rotating a single polarizer and studied the polarization De-Glints method using DoFP-visible polarimeters in this section.

### 2.1. Principles of Polarization Imaging

Polarized light is generally described by the Stokes vector *S*, which can be expressed as

(1) $$\;S={\left[\begin{array}{cccc} S_{0} & S_{1} & S_{2} & S_{3} \end{array}\right]}^{T}$$

where $S_{0}$ is the total light intensity, $S_{1}$ is the horizontal or vertical linear polarization component, $S_{2}$ is the linear polarization component of +45° or −45°, and $S_{3}$ is the left- or right-handed circular polarization component.

When examining the polarization state of a scene, two parameters are typically used: the DoP and AoP. The DoP represents the proportion of the polarization component in the total intensity. The AoP represents the angle between the direction of the maximum polarization energy in the incident light and the *x*-axis of the reference coordinate system. Normally, the circular polarization condition ($S_{3}\neq 0$) is rarely satisfied when light is scattered in the atmosphere or reflected from a water surface [30]. Supposing $S_{3}=0$, according to the Stokes vector *S*, the equation for calculating DoP and AoP is expressed as

(2) $$\;DoP=\frac{\sqrt{S_{1}^{2}+S_{2}^{2}}}{S_{0}},AoP=\frac{1}{2}\arctan\left[\frac{S_{2}}{S_{1}}\right]$$

The Stokes vector of arbitrary light can be represented by DoP and AoP as follows:

(3) $$\;S=S_{0}\left[\begin{array}{c} 1\\ DoP\cos(2AoP)\\ DoP\sin(2AoP)\\ 0 \end{array}\right]$$

The change in the polarization state caused by the optical element of the light wave is described by Mueller matrix *M*:

(4) $$\;S_{out}=\left[\begin{array}{c} {S_{0\_}}_{out}\\ {S_{1\_}}_{out}\\ {S_{2\_}}_{out}\\ {S_{3\_}}_{out} \end{array}\right]=M\cdot S_{in}=\left[\begin{array}{cccc} M_{11} & M_{12} & M_{13} & M_{14}\\ M_{21} & M_{22} & M_{23} & M_{24}\\ M_{31} & M_{32} & M_{33} & M_{34}\\ M_{41} & M_{42} & M_{43} & M_{44} \end{array}\right]\cdot \left[\begin{array}{c} {S_{0\_}}_{in}\\ {S_{1\_}}_{in}\\ {S_{2\_}}_{in}\\ {S_{3\_}}_{in} \end{array}\right]$$

where $S_{in}$ is the incident Stokes vector, $S_{out}$ is the output Stokes vector, and *M* is the Mueller matrix of an optical element that represents the effect of an optical element or system on the incident light.

The photoelectric imaging device can only respond to light intensity; thus, the total light intensity is expressed as

(5) $$\;S_{0\_out}=M_{11}\cdot S_{0\_in}+M_{12}\cdot S_{1\_in}+M_{13}\cdot S_{2\_in}+M_{14}\cdot S_{3\_in}$$

The Stokes vector of incident radiation can be calculated according to the light intensity values measured in four uncorrelated directions. Subsequently, the polarization information of the incident radiation, such as the DoP and AoP, can be obtained.

### 2.2. Traditional Glint Suppression Method by Polarization

The light reflected from the water surface exhibits high intensity and obvious polarization characteristics. The objects on or under the water usually have low intensity without polarization characteristics in the natural scenes. We denote the light with and without polarization characteristics as $I_{p}$ and $I_{up}$, respectively. Thus, the original image *I* can be represented as

(6) $$\;I=I_{p}+I_{up}$$

According to Marius’s law, the intensity of the incident light after passing through the polarizer becomes

(7) $$\;I\left(\theta \right)=I_{p}\left(\theta \right)+\frac{1}{2}I_{up}=I_{p}\cos^{2}\left(\partial -\theta \right)+\frac{1}{2}I_{up}$$

where *∂* is the vibration direction of the linear polarization light and θ is the transmission direction of the linear polarizer.

The installation of a polarizer in front of the lens, with the transmission direction being orthogonal to the vibration of the linear polarized light, can eliminate the reflected polarized light and suppress glints [18]. In the experiment, the observation geometry was determined according to a theoretical model and the glint suppression direction was determined by rotating the polarizer [19]. To reduce the error caused by the manual rotation of the polarizer and to increase the speed, a stepper motor can be used [31]. Consequently, the polarization differential method was implemented. This method calculates DoP through two orthogonal polarized images:

(8) $$\;P=\frac{I_{\|}-I_{⊥}}{I_{\|}+I_{⊥}}$$

The polarization differential method combined with the scattering model has been widely used for image defogging and underwater de-scattering [32,33,34].

### 2.3. Automatic Glint Suppression Method Based on the DoFP-Visible Polarimeter

The DoFP polarimeter can simultaneously acquire images in four different polarization directions (0°, 45°, 90°, and 135°) of the scene. This is conducive to obtaining the polarization images of the dynamic scene in real time, calculating different parts of the image independently, solving the Stokes vector, and calculating the DoP and AoP. These outstanding advantages aid the DoFP polarimeter in overcoming the inherent defects of the DoT polarimeter.

The Stokes vectors $S_{0}$, $S_{1}$, and $S_{2}$ can be expressed as

(9) $$\;\begin{array}{c} S_{0}=I_{0}+I_{90}\\ S_{1}=I_{0}-I_{90}\\ S_{2}=I_{45}-I_{135} \end{array}$$

The general expression for the Mueller matrix $M_{p}$ of an ideal linear polarizer with a theoretical angle θ between the transmission and horizontal direction (*x*-axis) is expressed as follows:

(10) $$\;M_{p}=\frac{1}{2}\left(\begin{array}{cccc} 1 & \cos2\theta & \sin2\theta & 0\\ \cos2\theta & \cos^{2}2\theta & \sin2\theta \cos2\theta & 0\\ \sin2\theta & \sin2\theta \cos2\theta & \sin^{2}2\theta & 0\\ 0 & 0 & 0 & 0 \end{array}\right)$$

As the first row of $M_{p}$ is $M_{m}$, combining this with Equation (5) yields the light intensity after transmission through the polarizer as

(11) $$\;I_{\theta }=M_{m}\cdot S=\frac{1}{2}\left(S_{0}+S_{1}\cos2\theta +S_{2}\sin2\theta \right)$$

The images in each polarization direction can be derived using Equation (11). Combined with Equation (7), it is evident that the detection light intensity $I_{\theta }$ was related to the transmission direction θ of linearly polarized light. When θ was perpendicular to the vibration direction *∂* of polarized light, the polarization part was reduced to 0, the detection light intensity reached the minimum value and the glint was suppressed to the greatest extent.

The grayscale value of the image in the direction θ is $f_{\theta }\left(i,j\right)$. We define the average grayscale $\bar{f_{\theta }}$ as the index for evaluating the effect of the glints on $f_{\theta }\left(i,j\right)$:

(12) $$\;\bar{f_{\theta }}=\frac{1}{WH}\sum_{i=1}^{W}\sum_{j=1}^{H}f_{\theta }\left(i,j\right)$$

where *WH* refers to the image size.

If the image in the direction $\theta_{i}$ has the minimal average grayscale $\bar{f_{\theta i}}$, $\theta_{i}$ is the best polarization angle $\theta_{best}$ wherein the glints exert the minimum effect on the image.

(13) $$\;\begin{array}{c} \bar{f_{\theta i}}=min\left\{\bar{f_{\theta }}\right\}=min\left\{\bar{f_{0}},\bar{f_{1}},\cdots ,\bar{f_{180}}\right\}\\ \theta_{best}=\theta_{i} \end{array}$$

Therefore, the image at the best angle after polarization processing can be expressed as follows (the DoFP-14 method in brief):

(14) $$\;I_{\theta_{best}}=\frac{1}{2}\left(S_{0}+S_{1}\cos2\theta_{best}+S_{2}\sin2\theta_{best}\right)$$

In practice, the water surface is not perfectly still owing to the influence of moving objects and ambient winds. Thus, the polarization characteristics of each part are not the same. To obtain a better image effect, each pixel was solved via polarization. The Stokes vector at (i,j) is S(i,j) and the corresponding Muller matrix is $M_{m(i,j)}$; then, the light intensity after polarization processing is expressed as follows (the De-Glints method for short):

(15) $$\;I_{\theta (i,j)}=M_{m(i,j)}\cdot S\left(i,j\right)$$

## 3. Experiment

The aim of this study is to compare the effectiveness of glint suppression between DoT and DoFP systems. We use a PHX050S DoFP polarimeter (LUCID Vision Labs) with a sensor size of 2048 × 2448 px. The focal length is 25 cm. The image format adopted is 8 bits, and the max extinction ratio is 425:1. Simultaneously, a CM3-U3-13S2M monochrome ordinary camera with a sensor size of 960 × 1280 px is used to acquire polarized images with rotating polarizers as a DoT system. The polarizer extinction ratio is 800:1. The focal length is 12 cm. We record data on light intensity at the water surface using a hand-held digital light meter (Aicevoos AS-V10 Digital Light Meter) with a range of 0 to 200,000 lux. Its resolution is 0.1 lux for illuminances less than 1000 lux and 1 lux for illuminances larger than or equal to 1000 lux.

The DoFP system possesses a significant advantage in dynamic processing, rendering it the focal point of this study. However, the structure of its detector leads to crosstalk among adjacent pixels, which ultimately dilutes its accuracy in detecting polarization information when compared with the DoT system. Therefore, we investigate the accuracy, effectiveness, and adaptability of the DoFP system within the laboratory and in field scenarios.

**Figure 1:**
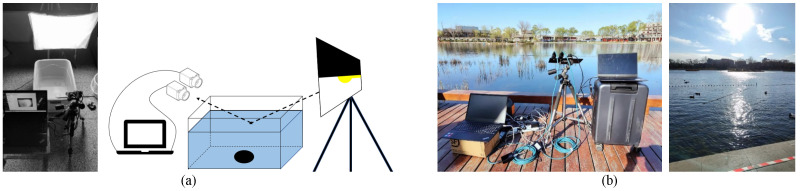
Experimental equipment and schematic diagram: (**a**) indoors and (**b**) outdoors.

The experimental scenario and equipment are shown in Figure 1. We perform nine sets of indoor experiments including six static scenes T1–T6 and three dynamic scenes V1–V3. Static experiments are used to verify accuracy and effectiveness. Dynamic experiments are used to verify adaptability. The details of the experiments are shown in Table 1. The light source for indoor experiments is a conventional white LED, which is placed in a highly reflective softbox for uniform illumination when in use. The water depth of the object and the light intensity on the water surface are recorded. The inclination of the light source for all nine experiments is 68° and the exposure time is 50 ms. The observation elevation angle indicates the angle between the camera’s optical axis and the water surface in the horizontal direction. We simulate static scenes and different dynamic scenes by controlling the wind to disturb the water surface at different speeds.

Outdoor experiments O1–O3 in three different locations are conducted in Shichahai, Beijing, to verify the practical utility, as shown in Figure 1b and Table 1. We regard the tank under the water where the lotus is cultivated as the main observation target. The water surface is constantly disturbed by winds from different directions.

## 4. Results and Discussion

To effectively handle dynamic scenes and overcome the limitations of the DoT system, the proposed DoFP-polarimeter-based glint suppression method is experimentally verified and discussed.

### 4.1. Criteria

To facilitate objective evaluation, the following indexes are used:

1. The accuracy of the angle calculation is described using the relative error δ
  (16) $$\;\delta =\frac{\theta_{m}-\theta_{0}}{\theta_{0}}\;\times 100\%$$
  where $\theta_{m}$ is the angle calculated from the data obtained via the DoFP polarimeter and $\theta_{0}$ is the angle obtained via the DoT polarimeter, which is considered to be the standard value.
2. We use the comprehensive image quality evaluation index *E* to evaluate image quality:
  (17) $$\;E=\frac{C\times G}{\sigma }\times 1000$$
  where *C* is the contrast, *G* is the mean gradient, and σ is the standard deviation.
  Contrast is defined as the intensity ratio of the target to the background:
  (18) $$\;C=\left|\frac{\mu_{t}-\mu_{b}}{\mu_{t}+\mu_{b}}\right|$$
  where $\mu_{t}$ and $\mu_{b}$ represent the average intensities of the target and local background around the target, respectively. The contrast between the target and background reflects the enhancement of the target. A higher contrast indicates a smaller impact of the glints and a better effect of the algorithm.
  Average gradient refers to the variation in the image detail grayscale values. A larger average gradient indicates richer detail.
  (19) $$\;G=\frac{1}{\left(M-1\right)\left(N-1\right)}\sum_{i=1}^{M-1}\sum_{j=1}^{N-1}\sqrt{\frac{1}{2}\left\{{\left(\frac{\partial f\left(i,j\right)}{\partial i}\right)}^{2}+{\left(\frac{\partial f\left(i,j\right)}{\partial j}\right)}^{2}\right\}}$$
  The standard deviation reflects the discrete condition of the image grayscale values:
  (20) $$\;\sigma =\sqrt{\frac{1}{MN}\sum_{i=1}^{M}\sum_{j=1}^{N}{\left(f\left(i,j\right)-\bar{f}\right)}^{2}}$$
  The original image is affected by the glints and has a high local grayscale value, which manifested as a large standard deviation. The extreme distribution of pixel grayscale values should be reduced after processing; therefore, the smaller the standard deviation, the better the method.
  The *E* indicator is proportional to the contrast *C* and average gradient *G* and inversely proportional to the standard deviation σ. Combined with the aforementioned analysis of the three indicators, the larger the value of *E*, the better the quality of the characterization image.
3. The relative improvement in the *E* index, δE, is used as an indicator to evaluate the effectiveness of the algorithm:
  (21) $$\;\delta E=\frac{E_{p}-E_{o}}{E_{o}}$$
  where $E_{p}$ and $E_{o}$ are the *E* indicator values of the processed and original images, respectively.

### 4.2. Comparison of DoT and DoFP Systems

**Figure 2:**
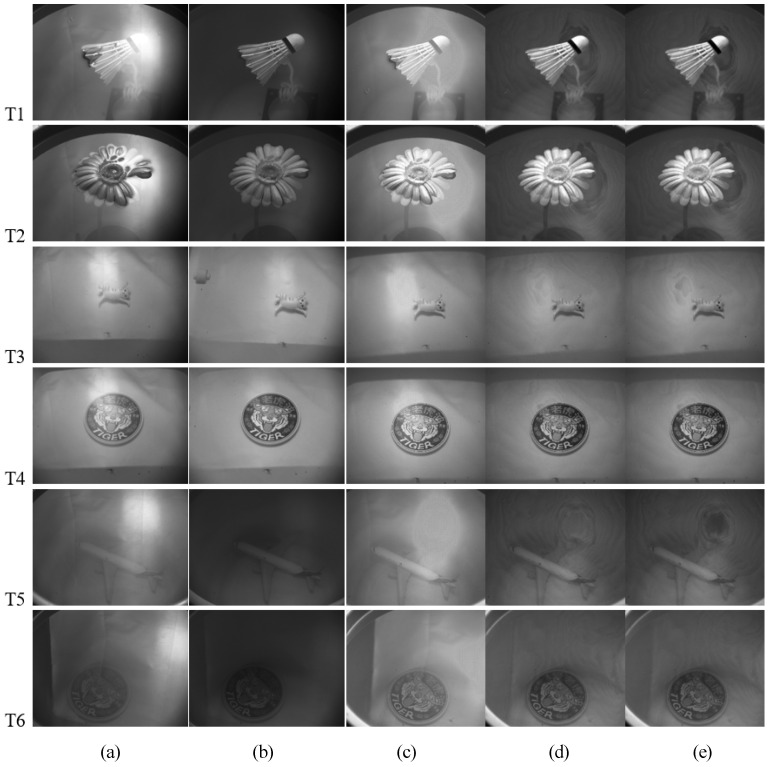
Static experimental images. (**a**) Original images obtained using a DoT polarimeter. (**b**) Polarization images obtained using a DoT polarimeter. (**c**) Original images obtained using a DoFP polarimeter. (**d**) Images calculated using DoFP-14. (**e**) Images calculated using De-Glints.

As shown in Figure 2, to verify the accuracy of the calculations, six sets of static experiments are performed indoors. Figure 2a shows the original images obtained using the DoT polarimeter. By rotating the polarizer of the DoT system, we obtained the images with minimal intensity as the polarization suppression results (shown in Figure 2b). As is evident, the glints are effectively suppressed after filtering by the polarizer, and the spots of the original image with a high gray value mostly disappeared. The difference in intensity between different parts of the target (above or below the water surface) in T1 and T2 is reduced and the image is more integral. Furthermore, the underwater objects in T3–T6 are clearer. The original images obtained by the DoFP polarimeter are shown in Figure 2c. The results of calculation using the DoFP-14 method are shown in Figure 2d. The glints are effectively suppressed and the details above and below the water surface are clearer. Regardless of a DoT or DoFP polarimeter, the images after polarization processing are all sharper than the original images.

The best polarization angles measured by the DoT polarimeter and calculated by DoFP-14 for the six scenes are presented in Table 2, respectively. The calculated results are different from the polarization angle obtained by rotating the polarizer. In fact, there are certain errors between the DoT measurements and the actual true values, and the relative errors here are only calculated to compare the differences between the DoFP and the DoT polarimeters. The data in the table show that of the six comparison scenes we selected, the deviation of the T2 group is the largest, and the relative errors of the remaining groups are within 10%, indicating that the best polarization angles calculated by the DoFP system and the DoT system are basically the same.

### 4.3. Static Results

Considering that the polarization direction is not the same everywhere on the water surface, we suppress the glints using De-Glints; the results are shown in Figure 2e. In the previous section, we discussed the remarkable effect of the polarization method in Figure 2a–d. Figure 2e also clearly shows that the processing results effectively suppressed the glints. However, it is difficult to subjectively judge whether there is an improvement compared to the previous method. The comparison is made through the evaluation indicators proposed in Section 4.1.

We calculate the *E* indices for a–e in T1–T6, as shown in Figure 2, and obtain the relative degree of enhancement δE after processing, which represents the degree of improvement in image quality. $\delta E_{1}$, $\delta E_{2}$, and $\delta E_{3}$ are the degrees of *E* index enhancement after DoT, DoFP-14, and De-Glints polarization processing, respectively. The results are presented in Table 3. As is evident, for all six groups of the experiment, the values of $\delta E_{3}$ are almost all bigger than $\delta E_{1}$ and $\delta E_{2}$, indicating that the results for De-Glints are better than those for DoFP-14 and DoT, which means that the De-Glints method is more effective.

### 4.4. Dynamic Results

According to the previous analysis, the DoT polarimeter is structurally destined to be inferior to the DoFP polarimeter for dynamic scenes. In this section, we perform three sets of dynamic experiments V1–V3 with different wind speeds and analyze the change in the best polarization direction. We judge the image quality using *E* as an evaluation index to verify the dynamic adaptive capability of the DoFP system.

**Figure 3:**
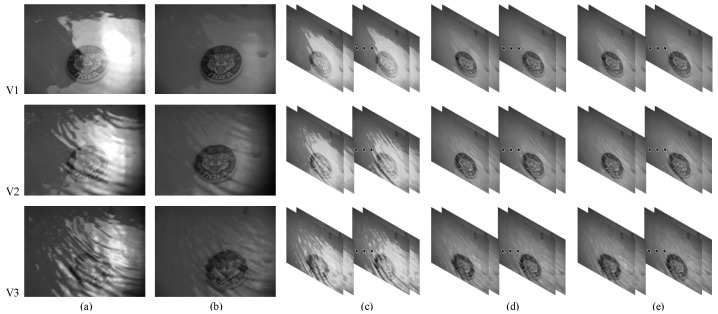
Dynamic experimental images. (**a**) Original images obtained using the DoT polarimeter. (**b**) Polarization images obtained using the DoT polarimeter. (**c**) Original images obtained using the DoFP polarimeter. (**d**) Images calculated using DoFP-14. (**e**) Images calculated using De-Glints.

The results are shown in Figure 3. Figure 3a shows the original images obtained by the DoT polarimeter. The DoT system cannot change the polarization direction of the polarizer in real time. Under the same illumination and geometric conditions, water surface disturbances can affect the polarization characteristics, which may change the best polarization direction. From Figure 3b, we can see there is also significant glint suppression in one fixed polarization direction, because of the attenuation of the light intensity by the polarizer. The polarized images showed that the surface reflections are suppressed and underwater targets are more clearly defined. The original images acquired by the DoFP polarimeter are shown in Figure 3c. Ten consecutive images of the dynamic scene were processed using DoFP-14 and De-Glints, as shown in Figure 3d,e, respectively. The glint is well suppressed and the image is considerably clearer.

**Figure 4:**
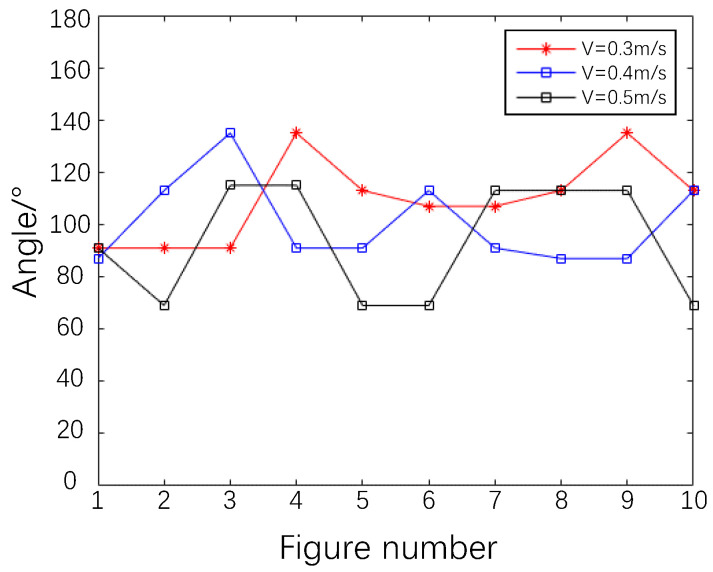
Values of the best polarization direction for 10 consecutive frames calculated using DoFP-14 at different wind speeds.

The variation in the best polarization angle calculated using DoFP-14 is shown in Figure 4. As is evident, in dynamic scenes, the best polarization angle fluctuates within a range with the water surface. With the increase in the wind speed, the water surface undulates faster and more dramatically, and the relative change in the best polarization angle increases. Furthermore, the DoT polarimeter is affected to a large extent by the changes in water surface conditions, which is difficult to overcome. For the DoFP system, the best polarization angle can be calculated separately for each frame in real time, such that the DoFP-14 and De-Glints are not affected by changes in the best polarization direction. In dynamic scenes, the DoFP polarimeter exhibits outstanding superiority.

**Figure 5:**
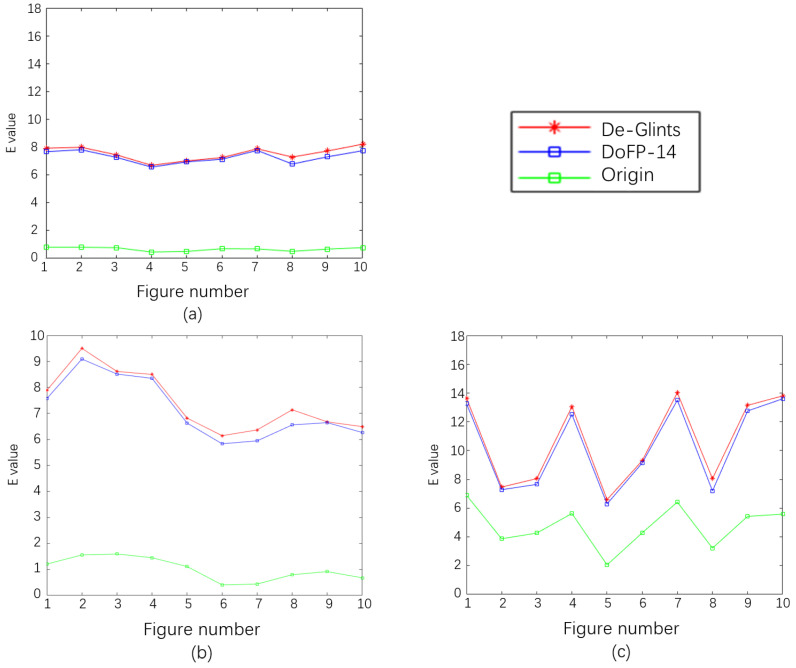
Results of image quality *E* index evaluation: (**a**) 0.3 m/s; (**b**) 0.4 m/s; (**c**) 0.5 m/s.

The *E* indices of the original, DoFP-14, and De-Glints images of the water surface in V1–V3 are shown in Figure 5. After polarization processing, the *E* index increased significantly, representing a significant improvement in image quality. The results of DoFP-14 and De-Glints are close, but those of De-Glints are slightly better.

### 4.5. Outdoor Results to Verify Practical Utility

**Figure 6:**
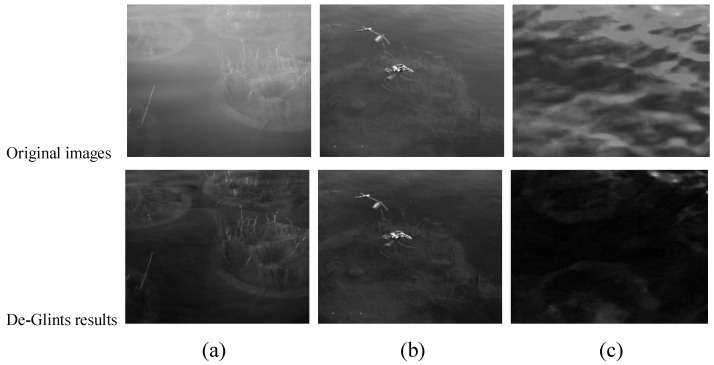
Effect of De-Glints in real scenes: (**a**) O1; (**b**) O2; (**c**) O3.

As shown in Figure 6, we chose three different locations for our experiments, O1–O3. It is evident that, in the actual scene, the glint has a strong interference with the underwater target, blurring the details of the target and introducing interference, or limiting the integration time of the image to prevent overexposure owing to the high intensity of the glint. This results in low gray values of the target, which are difficult to distinguish for the human eye. In the De-Glints results, the effect of the glints is reduced. The comparison in Figure 6 shows that the glints are largely suppressed and the underwater scenes are displayed more clearly after De-Glints processing. In the case of very heavy surface flare effects (Figure 6c), the target details in the water in the original image are completely drowned out. After processing, the glint is well suppressed and the underwater target is faintly visible.

The evaluation indicators of the images in Figure 6 are shown in Table 4. The *E* indicators of the De-Glints results are all positively enhanced. In the extreme case of O3, the δE is 8.00. This result shows that the information about water clutter and glints in the processed images is suppressed and that the underwater targets are enhanced, proving the effectiveness of the method in practical scenarios.

**Figure 7:**
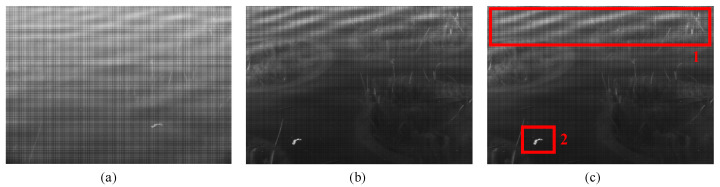
Images obtained using the DoT polarimeter in O1. (**a**) Original image obtained using the DoT system. (**b**) Polarization image obtained using the DoT system. (**c**) Areas of interest. Rectangle 1: Obvious residual glint; Rectangle 2: Dynamic object.

As shown in Figure 2b, the DoT polarimeter also shows an effective performance in glint suppression. In outdoor experiments, however, it shows obvious limitations. The original image obtained using the DoT polarimeter in O1 is shown in Figure 7a. The polarization image is shown in Figure 7b. We mainly wish to discuss two points of interest, marked by red rectangles in Figure 7c. In polarized images, the glint is unevenly suppressed in each region. In rectangle 1, we see that the residual glint is very obvious in the marginal region, whereas it is suppressed well in the DoFP results, as shown in Figure 7a. During the experiment, when we rotated the filter to find the best polarization direction, the position of the floating object on the water surface in rectangle 2 moved significantly, which was a situation in the DoFP system that could be comfortably avoided.

### 4.6. Enhancement

**Figure 8:**
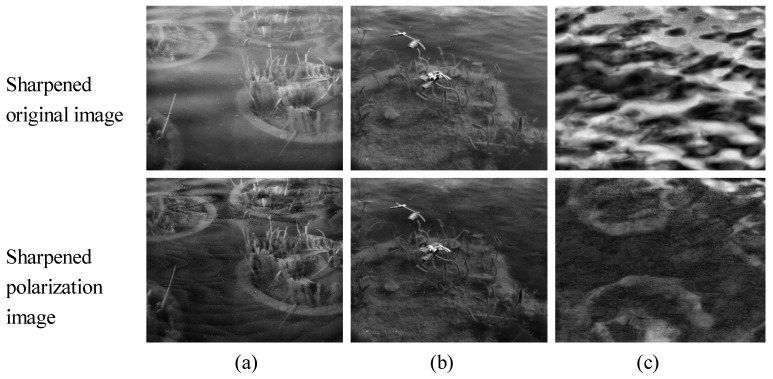
Image enhancement: (**a**) O1; (**b**) O2; (**c**) O3.

Whether it is a DoT or DoFP system, after polarization detection, the polarized image will be darker than the intensity image. Image-enhancement techniques can improve the image intensity level and distribution, and help the subjective judgment of image quality. We take the histogram equalization method as an example. Figure 8 shows the enhanced original and polarized images in O1–O3, respectively. As evident from the figures, the effect of water surface glints in the original image is amplified following processing with the superimposed enhancement algorithm, which seriously affected the observation of underwater scenes. For the polarized image, the targets are well enhanced. In the case of very heavy surface flare effects (Figure 8c), the underwater target details in the original image are completely drowned out. Following histogram equalization, glints and ripples are magnified and the underwater target is completely invisible. After De-Glints processing, the glint is well suppressed and the underwater target is faintly visible, and then after equalization, the outline of the underwater object is clearly shown. Thus, polarization imaging is very effective in the suppression of surface glint and makes a significant contribution to subsequent enhancement algorithms, even recognition algorithms.

However, certain areas of this study require improvement, and the accuracy of the polarization direction calculation and the overall homogeneity of the results will be further optimized in the future. The method described in this study is based on the principle of polarization imaging, and the intensity of the results after polarization filtering is generally low. Thus, the superimposed histogram equalization method in this study is only a small attempt to visualize the results, and how better to design the algorithm is something that needs to be studied.

## 5. Conclusions

In this study, we present De-Glints, a method for suppressing solar glints and obtaining clear underwater images through the water surface based on a DoFP-visible polarimeter. This method is dependent on the significant partial polarization characteristics of the glints. The DoFP-visible polarimeter is used to obtain the characteristics of different polarization directions simultaneously, perform polarization measurements on the water surface disturbed by the glints, create polarization radiation maps in four polarization directions, and calculate the Stokes parameters. Based on the principle of polarization imaging, the best polarization angle and the image corresponding to the minimum average grayscale of each pixel are calculated to achieve the suppression of glints. In this study, index *E* is designed to evaluate the degree of image quality improvement. We examine the accuracy, effectiveness, and adaptability of the proposed method using the DoFP system while also validating its practical utility in real-world scenarios.

The results of indoor and outdoor experiments show that the error in the selection of the best polarization angle using DoFT calculations is essentially within 10% compared to the DoT polarimeter. The minimum error is only 3%. The proposed methods, DoFP-14 and De-Glints, improve the image quality significantly in both evaluation indices, *E*; however, De-Glints works a little better. The DoFP polarimeter demonstrates significant superiority in terms of adapting to dynamic scenes. In addition, the results of the outfield experiment also prove that, in the case of strong flare interference, the glint is effectively suppressed following polarization processing, and the *E* index could also be relatively improved by 8.00. The details and contour information of the target in the water body are clearer, which proves the correctness and necessity of the method in this study.

The proposed method can be applied to improve the detection, identification, and tracking of targets in water. Thus, using the target polarization information to effectively suppress flare interference and improve the contrast of the target has important practical application significance.

## Figures and Tables

**Table 1:** Parameters of indoor and outdoor experiments.

Scenes	Depth/cm	Light Intensity/Lux	Observation Angle	Wind Speed/m·s^−1^
T1	0	610	39°	0
T2	0	610	39°	0
T3	5.6	425	29°	0
T4	5.6	425	29°	0
T5	22	610	35°	0
T6	22	610	35°	0
V1	15	440	39°	0.3
V2	15	440	39°	0.4
V3	15	440	39°	0.5
O1	≈100	24,000	30.7°	-
O2	≈100	34,400	25.9°	-
O3	≈150	83,200	35°	-

**Table 2:** Best polarization angles and relative errors between DoT and DoFP polarimeters.

	T1	T2	T3	T4	T5	T6
DoT$\theta_{\mathrm{best}}$	103°	100°	116°	116°	96°	97°
DoFP$\theta_{\mathrm{best}}$	110°	88°	113°	113°	105°	93°
δ	7%	12%	−3%	−3%	9%	4%

**Table 3:** Image quality enhancement using different polarization processing methods.

*E*	T1	T2	T3	T4	T5	T6
a	6.42	5.20	3.07	3.08	1.53	11.15
b	20.04	19.24	3.46	9.07	5.60	13.90
$\delta E_{1}$	2.12	2.70	0.13	1.95	2.65	0.25
c	2.86	3.05	0.09	1.48	0.07	4.56
d	14.88	12.55	1.43	4.75	3.93	5.82
$\delta E_{2}$	4.20	3.11	14.64	2.20	58.87	0.28
e	15.05	13.30	1.44	5.07	4.13	5.84
$\delta E_{3}$	4.26	3.36	14.72	2.42	61.91	0.28

**Table 4:** Results of the outfield experiment.

		*C*	*G*	σ	*E*
O1	Original image	0.02	0.75	37.24	0.34
	De-Glints	0.24	1.42	17.04	19.64
O2	Original image	0.05	0.55	21.78	1.21
	De-Glints	0.07	0.79	16.05	3.23
O3	Original image	0.23	0.80	29.98	6.21
	De-Glints	0.56	1.11	11.11	55.91

## Data Availability

Not applicable.

## Abbreviations

The following abbreviations are used in this manuscript:

DoFP	Division of Focal Plane
DOT	Division of Time
DoP	Degree of Polarization
AoP	Angle of Polarization

## References

[1] Rocher J., Jimenez J.M., Tomas J., Lloret J. (2023). Low-Cost Turbidity Sensor to Determine Eutrophication in Water Bodies. Sensors.

[2] Jang A., Zou Z., Lee K.K., Ahn C.H., Bishop P.L. (2011). State-of-the-art lab chip sensors for environmental water monitoring. Meas. Sci. Technol..

[3] Mohindru P. (2022). Development of liquid level measurement technology: A review. Flow Meas. Instrum..

[4] Ogilvie A., Belaud G., Massuel S., Mulligan M., Le Goulven P., Calvez R. (2018). Surface water monitoring in small water bodies: Potential and limits of multi-sensor Landsat time series. Hydrol. Earth Syst. Sci..

[5] Jian M., Liu X., Luo H., Lu X., Yu H., Dong J. (2021). Underwater image processing and analysis: A review. Signal Process. Image Commun..

[6] Samann F.E. (2018). Real-time liquid level and color detection system using image processing. Acad. J. Nawroz Univ..

[7] Zhang Z., Zhou Y., Liu H., Gao H. (2019). In-situ water level measurement using NIR-imaging video camera. Flow Meas. Instrum..

[8] Lynch D.K., Dearborn D.S., Lock J.A. (2011). Glitter and glints on water. Appl. Opt..

[9] Shaw J.A. (1999). Degree of linear polarization in spectral radiances from water-viewing infrared radiometers. Appl. Opt..

[10] Wang G., Wang J., Zhang Z., Cui B. (2016). Performance of eliminating sun glints reflected off wave surface by polarization filtering under influence of waves. Optik.

[11] Lv Y., Sun Z., Zhao Y. (2012). Measurement of water-leaving radiance on smooth water surfaces at different viewing angles using high-resolution spectroradiometer. Chin. Opt. Lett..

[12] Mobley C.D. (2015). Polarized reflectance and transmittance properties of windblown sea surfaces. Appl. Opt..

[13] Hieronymi M. (2016). Polarized reflectance and transmittance distribution functions of the ocean surface. Opt. Express.

[14] Chami M., McKee D. (2007). Determination of biogeochemical properties of marine particles using above water measurements of the degree of polarization at the Brewster angle. Opt. Express.

[15] D’Alimonte D., Kajiyama T. (2016). Effects of light polarization and waves slope statistics on the reflectance factor of the sea surface. Opt. Express.

[16] Zibordi G. (2016). Experimental evaluation of theoretical sea surface reflectance factors relevant to above-water radiometry. Opt. Express.

[17] Sun Z., Wu D., Lv Y. (2022). Effects of water salinity on the multi-angular polarimetric properties of light reflected from smooth water surfaces. Appl. Opt..

[18] Zheng J., Zhao H., Li Y., Cheng C., Sun X., Song P., Wang S. (2017). Target detection in sun glint using the improved MWIR polarization technique. Proceedings of the Infrared Sensors, Devices, and Applications VII.

[19] Dolin L., Turlaev D. (2020). Polarization method for imaging through the water surface. Appl. Opt..

[20] Shaw J.A., Vollmer M. (2017). Blue sun glints on water viewed through a polarizer. Appl. Opt..

[21] Harnett C.K., Craighead H.G. (2002). Liquid-crystal micropolarizer array for polarization-difference imaging. Appl. Opt..

[22] Gilerson A., Carrizo C., Ibrahim A., Foster R., Harmel T., El-Habashi A., Lee Z., Yu X., Ladner S., Ondrusek M. (2020). Hyperspectral polarimetric imaging of the water surface and retrieval of water optical parameters from multi-angular polarimetric data. Appl. Opt..

[23] Zhao H., Ji Z., Zhang Y., Sun X., Song P., Li Y. (2016). Mid-infrared imaging system based on polarizers for detecting marine targets covered in sun glint. Opt. Express.

[24] Farlow C.A., Chenault D.B., Pezzaniti J.L., Spradley K.D., Gulley M.G. (2002). Imaging polarimeter development and applications. Polarization Analysis and Measurement IV.

[25] Tyo J.S. (2006). Hybrid division of aperture/division of a focal-plane polarimeter for real-time polarization imagery without an instantaneous field-of-view error. Opt. Lett..

[26] Perkins R., Gruev V. (2010). Signal-to-noise analysis of Stokes parameters in division of focal plane polarimeters. Opt. Express.

[27] Gruev V., Perkins R., York T. (2010). CCD polarization imaging sensor with aluminum nanowire optical filters. Opt. Express.

[28] Takruri M., Abubakar A., Alnaqbi N., Al Shehhi H., Jallad A.H.M., Bermak A. (2020). DoFP-ML: A machine learning approach to food quality monitoring using a DoFP polarization image sensor. IEEE Access.

[29] Chang J., He H., He C., Ma H. (2016). DofP polarimeter based polarization microscope for biomedical applications. Proceedings of the Dynamics and Fluctuations in Biomedical Photonics XIII.

[30] Zhou G., Xu W., Niu C., Zhao H. (2013). The polarization patterns of skylight reflected off wave water surface. Opt. Express.

[31] Liang J.A., Wang X., Fang Y.J., Zhou J.J., He S., Jin W.Q. (2018). Water surface-clutter suppression method based on infrared polarization information. Appl. Opt..

[32] Schechner Y.Y., Narasimhan S.G., Nayar S.K. (2001). Instant dehazing of images using polarization. Proceedings of the 2001 IEEE Computer Society Conference on Computer Vision and Pattern Recognition, CVPR 2001.

[33] Schechner Y.Y., Karpel N. (2005). Recovery of underwater visibility and structure by polarization analysis. IEEE J. Ocean. Eng..

[34] Treibitz T., Schechner Y.Y. (2008). Active polarization descattering. IEEE Trans. Pattern Anal. Mach. Intell..

